# Middle East Deployment and Lymphoid Malignancies in US Veterans: A Matched Case-Control Analysis

**DOI:** 10.3390/cancers17193161

**Published:** 2025-09-29

**Authors:** Helen Ma, Pankaj Gupta

**Affiliations:** 1 VA Long Beach Healthcare System, Long Beach, CA 90822, USA; pankaj.gupta@va.gov; 2 University of California, Irvine, Orange, CA 92868, USA; 3 UCI Chao Family Comprehensive Cancer Center, Irvine, CA 92868, USA

**Keywords:** lymphoma, multiple myeloma, lymphoid leukemia, outcomes research

## Abstract

**Simple Summary:**

The United States was in several conflicts in the Middle East spanning decades with potential military exposures resulting in long-term health effects. Many veterans involved in these operations reported high levels of environmental exposures and concerns about health effects, including blood cancers such as lymphoid malignancies, related to exposures including depleted uranium and burn pits. The aim of our retrospective matched case-control study was to assess the association between deployment and the risk of developing lymphoid malignancies. Our study did not find an increased risk of developing lymphoid malignancies, highlighting the need for prospective studies on military exposures.

**Abstract:**

**Background/Objective:** US military personnel deployed to the Middle East were potentially subjected to harmful exposures, such as carcinogens from burn pits, which may increase the risk of lymphoid malignancies. Our objective was to determine the association between deployment and the risk of developing lymphoid malignancies. **Methods:** This was a retrospective nested matched case-control study from a cohort of 3.5 million veterans who enlisted in the military after September 2001 and were followed until death or last follow up through September 2024. Cases of lymphoid malignancies were identified by the VA Central Cancer Registry and controls were randomly selected from the same base cohort, matched by year of birth, year of enlistment, sex, race, and ethnicity. Exposure was defined as deployment to the Middle East as determined by identification on the VA Operation Enduring Freedom/Operation Iraqi Freedom (OEF/OIF) roster with confirmed dates of deployment or paystubs. **Results:** There were 1037 cases of lymphoid malignancies and 3572 matched controls. Deployment was not associated with a higher risk of developing lymphoid malignancies compared to non-deployment. Exposure to OEF/OIF was not associated with a higher risk of developing certain types of lymphoid malignancies. **Conclusions:** In this large, matched case-control study of US veterans, deployment to the Middle East was not associated with increased risk of developing lymphoid malignancies. While these findings do not support an increased lymphoid malignancy risk, important limitations remain, including the absence of detailed exposure and potential confounding variables. Prospective monitoring of specific types and doses of exposures during military deployment, development of lymphoid and other malignancies, and their underlying pathophysiology is indicated.

## 1. Introduction

United States military personnel were deployed to the Middle East for several decades, raising concerns for potential long-term health effects, including lymphoid malignancies. Deployment to Afghanistan, known as Operation Enduring Freedom (OEF), and to Iraq, known as Operation Iraqi Freedom (OIF), from 2001–2014 was associated with harmful exposures, such as air pollution consisting of geologic dust, depleted uranium, improvised explosive devices, dioxins, and smoke from open burn pits, which were open airfields as large as 10 acres used to dispose of waste [1]. In addition, military personnel were also exposed to extreme weather, physical and psychological effects of combat, and limited nutrition [1,2,3]. Other military exposures, such as Agent Orange (which was contaminated with a potent dioxin) and radiofrequency radiation, have been found to be associated with the development of chronic conditions and cancers, including lymphoid malignancies [4,5,6]. Agent Orange exposure, however, was not associated with shorter survival in long term survivors with lymphoid malignancies [7]. Given the history of health effects resulting from military service, it is understandable that veterans deployed in OEF/OIF reported concerns about health effects related to potential exposures, such as depleted uranium, air pollution, and contaminated food and water [8].

Burn pits, associated with dioxins, polycyclic aromatic hydrocarbons, and other toxicants may be the most notorious exposure of the Middle East [9]. Chronic health conditions, such as post-traumatic stress disorder, headaches, pulmonary disease, and hypertension, are associated with exposure to open burn pits [3,10,11,12,13]. Previously, healthy military service personnel who went to the Middle East returned with respiratory symptoms associated with histopathologic changes in the lungs, highlighting the need to understand the chronic effects of inhalational exposures [14]. The composition of air that was sampled at military bases, however, was highly heterogeneous, limiting the ability to study specific toxicants and associated health effects [15]. Therefore, exposure assessments were dichotomized to broader determinations, such as deployment due to the lack of detailed data on dose and duration of specific carcinogens. This makes studying burn pit exposure and the association with cancers, such as lymphoid malignancies, difficult.

The little existing evidence that is available is mixed regarding whether deployment to the Middle East is associated with an increased risk of lymphomas [16,17,18]. In active military personnel who developed lymphoma, they did not appear to have shorter lifespans compared to a civilian population with lymphoma [19,20]. However, there was only a modest increased risk between exposure to burn pits and overall mortality, which was not driven by cancer or cardiovascular disease [21]. To the best of our knowledge, we conducted the largest nested case-control study from a cohort of 3.5 million veterans who enlisted after September 2001, with up to 13 years of follow-up, to determine whether deployment to the Middle East is associated with the risks of developing lymphoid malignancies.

## 2. Materials and Methods

### 2.1. Study Design

This was a nested matched case-control study of veterans enrolled in the United States Department of Veteran Affairs (VA) whose medical records were available using the VA Informatics and Computing Infrastructure (VINCI) [22]. Military exposure databases queried included the VA/Department of Defense Identity Repository (VADIR), the Individual Longitudinal Exposure Record (ILER), and the Airborne Hazards and Open Burn Pit Registry but these databases did not link to VA medical records to confirm lymphoid malignancy diagnoses and were not included [23,24,25]. The base cohort included veterans who enlisted in the military after 11 September 2001. The cohort was followed until date of death, date of last follow up, or the study cut-off date (30 September 2024). Cases of lymphoid malignancies were identified using the VA Central Cancer Registry (VACCR) using the International Code of Diseases, Oncology, Version 3 (ICD-O-3) [26]. Subtypes of lymphoid malignancies included acute lymphoblastic leukemia/lymphoma (ALL), Burkitt lymphoma (BL), chronic lymphocytic leukemia/small lymphocytic lymphoma (CLL/SLL), diffuse large B-cell lymphoma (DLBCL), follicular lymphoma (FL), Hodgkin lymphoma (HL), lymphoplasmacytic lymphoma (LPL), mantle cell lymphoma (MCL), marginal zone lymphoma (MZL), mature T/NK-cell lymphoma (MTNKL), lymphoma not otherwise specified (NOS), and plasma cell neoplasm (PCN). Given bimodal distribution of Hodgkin lymphoma, those younger than 40 at diagnosis were labeled as Adolescent and Young Adult (HL-AYA) and separated from those diagnosed at 40 years and older (HL-older) [27]. Incident cases were confirmed by ensuring the date of initial enlistment was earlier than the date of diagnosis. Controls were individuals without a known lymphoid malignancy.

### 2.2. Exposure Definition

The exposure of interest was deployment to the Middle East. Granular exposure data, such as base location, burn pit proximity, duration of exposure, were not available to link with clinical data. Therefore, the measure of exposure was dichotomized to OEF/OIF Service, which contained the list of veterans who served OEF and/or OIF with the dates indicating deployment or paystubs depending on the source [22]. Categorization of exposure was grouped as “Yes” or “No” because the military exposure assessment in the Middle East lacked dose measurements. Since dates of deployment were available, the latency from date of first deployment to diagnosis was calculated. Patients with a diagnosis of lymphoid malignancy prior to deployment were considered nondeployed for purposes of this analysis.

### 2.3. Data Collection and Statistical Methods

Demographic data, including sex, race and ethnicity, age at diagnosis of lymphoid malignancy (or index age for controls), were collected and used to match cases and controls [26]. Age at lymphoid malignancy diagnosis was calculated from date of birth and date of diagnosis for the cases. For the controls, their age at index date was calculated from date of birth and date of diagnosis for the corresponding matched cases. Each case was randomly matched (using a random number generator) with up to 4 controls, by year of birth (within 1 year), year of enlistment (within 1 year), sex, as well as race and ethnicity. Variables that potentially were effect measure modifiers included military branch and military rank. Military branch consisted of Air Force, Army, Marine Corps, Navy, Other (including Coast Guard, National Oceanic and Atmospheric Administration, U.S. Public Health Service). Military rank was divided among (1) enlisted personnel, who have a high school diploma or general equivalency diploma, (2) officers and warrant officers, who have college degrees or advanced technical training, respectively, and completed additional officer training, or (3) unknown. Categorical variables were compared using the chi-square test and continuous variables, such as the duration of enlistment (which was not normally distributed) was compared using the Wilcoxon rank-sum test. Time at risk was calculated from enlistment date to date of death or last follow up (30 September 2024). Veterans were excluded if there was no indication they had died and the date of last follow up was unknown.

For the analysis, the odds ratios (OR) and 95% confidence intervals (95% CI) were calculated to assess the strength of association between deployment to the Middle East and the development of any lymphoid malignancy and any of the more common subtypes (CLL/SLL, DLBCL, HL-AYA, MTNKL, or PCN). Conditional logistic regression was used to correct for matching to account for the fact that cases and controls were grouped based on year of birth, year of enlistment, sex, and race and ethnicity. A *p*-value of <0.05 (2-tailed) was considered statistically significant. Results were adjusted by military branch and rank. Analyses were performed using the Statistical Analysis System Enterprise Guide 8.3.

Sensitivity analyses were performed excluding cases diagnosed within 2, 5, and 10 years from deployment (including the corresponding controls) to account for latency of lymphomagenesis in the dataset using the VA OEF/OIF roster.

### 2.4. Ethics Statements

This study was approved by the Institutional Review Boards (IRB) at the VA Long Beach Healthcare System and the University of California, Irvine. A waiver of consent was approved by the IRB. Research was performed in accordance with the Declaration of Helsinki.

## 3. Results

### 3.1. Patient Characteristics

There were 1037 cases and 3572 matched controls included in the analysis. Patient characteristics are summarized in Table 1. Cases had a median age of 35.2 years (IQR 28.7–45.2) at diagnosis of lymphoid malignancy and were mostly men (*n* = 3616, 87%). Matched controls had a similar median age of 33.5 years (IQR 27.9–41.9) and similarly predominantly male (*n* = 4032, 87%). The majority were non-Hispanic White veterans (60%) followed by non-Hispanic Black (15%) and Hispanic (13%) veterans, similar in both cases and controls. The most common lymphoid malignancy subtypes were HL-AYA (*n* = 261, 25%), DLBCL (*n* = 146, 14%), and PCN (*n* = 114, 11%).

### 3.2. Military Characteristics and Exposure

Differences between cases and controls were noted in military variables of interest as summarized in Table 2. More than half of the cases and controls were in the Army (58% and 60%, respectively) and the rest were evenly distributed in the other branches, *p* = 0.60. There were similar enlisted personnel in the cases compared to the controls (89% vs. 87%), *p* = 0.08. In this case-control analysis, cases were just as likely to have been deployed compared to controls, *p* = 0.89.

### 3.3. Association of Deployment with Development of Lymphoid Malignancies

We conducted logistic regression analyses to examine the association between deployment and the development of lymphoid malignancies.

Deployment to OEF/OIF was not associated with a higher risk of developing lymphoid malignancies compared to nondeployment (Table 3). The OR was 1.05 (95% CI 0.89–1.23). To assess the latency in the risk of developing lymphoid malignancies of those deployed compared to nondeployed, we performed sensitivity analyses excluding cases diagnosed within 2, 5, and 10 years of initial deployment. With the exclusion of lymphoid malignancies diagnosed up to the first 2 years from deployment, the result was similar to the whole group (OR 0.95, 95% CI 0.81–1.12). In contrast to the results of the 2-year sensitivity analysis, when we excluded lymphoid malignancies diagnosed up to 5 or 10 years from first deployment, the risk of developing lymphoid malignancies in the deployed group was lower than in the nondeployed group (5-year sensitivity analysis: OR 0.75 (95% CI 0.64–0.90, *p* = 0.0017) and 10-year sensitivity analysis: OR 0.41 (95% CI 0.34–0.50, *p* < 0.0001).

### 3.4. Association of Deployment with Development of Individual Subtypes of Lymphoid Malignancies

Given the heterogeneity of lymphoid malignancies, we analyzed the association between deployment and the development of more common subtypes of lymphoid malignancies (Table 4). We found that exposure was not associated with increased risk of CLL/SLL, DLBCL, HL-AYA, MTNKL, or PCN any time after deployment. We also performed a sensitivity analysis excluding cases diagnosed at 2, 5, and 10 years for each subtype. When excluding cases diagnosed at 2 years after deployment, deployment was not associated with a difference in the risk of developing any of these subtypes. When excluding cases diagnosed up to 5 years after deployment, there was a lower risk of developing DLBCL and HL-AYA in the deployed group (OR 0.56 [95% CI 0.35–0.89], *p* = 0.01 and OR 0.67 [95% CI 0.47–0.94], *p* = 0.02, respectively). When excluding cases diagnosed up to 10 years after deployment, there was decreased risk in the development of all the 5 subtypes analyzed (see Table 4).

## 4. Discussion

We examined the association between deployment of US military personnel to the Middle East after September 11 2001 and the subsequent risk of development of lymphoid malignancies. Deployment was not associated with a higher risk of developing lymphoid malignancies in veterans who enlisted in the military after 11 September 2001 and enrolled in the VA compared to those who never deployed. Similarly, deployment was not associated with developing any of the five common subtypes (CLL/SLL, DLBCL, HL-AYA, MTNKL, and PCN) in the whole cohort.

In our sensitivity analyses, which excluded cases diagnosed up to 2, 5, and 10 years after deployment, we found no difference at 2 years but lower risk of developing lymphoid malignancies overall at 5 and 10 years. Our data suggest that lymphoid malignancies that develop late after initial Middle East deployment may not be related to the carcinogenic exposures that they may have experienced during deployment. When analyzing by subtypes to account for biologic differences in the individual lymphoid malignancies, the findings were broadly similar. Other investigators speculated whether similar or lower risks of developing lymphoid malignancies in military personnel may be in part due to a healthy soldier effect [27].

Ours is the first study, to the best of our knowledge, to examine the risk of lymphoid malignancies in a large cohort of US veterans deployed to the Middle East after 11 September 2001. Previous relevant reports have included either small numbers of personnel with lymphoid malignancies or did not examine the risk of development of lymphoid malignancies in deployed versus nondeployed personnel. One study of British military service personnel who deployed to the Gulf War compared to those who did not deploy reported that there was no increased risk of any type of cancer after follow-up through July 2002; however, this study included only 79 cases of lymphoid malignancies [28]. Another study reported that US active-duty service members (ADSM) who were deployed to the Middle East and reported burn pit exposure included only five cases of lymphoid malignancies, insufficient to determine any association between deployment and risk of developing lymphoid malignancies [18]. Sgrignoli et al. found that military personnel deployed to the Middle East were more likely to have aggressive subtypes of lymphoid malignancies compared to personnel who never deployed [16]. This study did not report on the risk of developing lymphoid malignancies in deployed and undeployed personnel.

Limitations in our retrospective matched case control analysis include selection bias, information bias, measurement bias, and confounding. There is potential selection bias as the cohort is limited to veterans who enlisted in the VA, since there may be those who enlisted and received their healthcare elsewhere. However, the cases and controls were chosen from the same base population to limit selection bias, restricting to the VA population. There is a potential for measurement bias because the deployment to the Middle East was dichotomized given absence of any method to determine the actual dose of individual toxicants. The pathogenesis of cancer is complex with multifactorial contributions in the mechanism of disease as well as varying latency between exposure and cancer development, so attribution of any one exposure is difficult. Data on other effect modifiers, such as tobacco and alcohol use, were limited to cancer cases in the VACCR and not available for controls. Lifestyle factors, including tobacco use, alcohol use, and BMI, are variably associated with the risk of developing different lymphoid malignancy subtypes so adjustment may not be relevant for certain subtypes [29,30,31]. For the current analysis, these variables, including the details such as cigarette pack years and amount of daily alcohol use, were not available so the analysis could not be adjusted for them.

To address these limitations, we performed a sensitivity analysis to explore the effect of latency between exposure and developing lymphoid malignancies. Excluding cases that developed up to 2 years after initial deployment did not change the finding that there was no difference in the risk of developing lymphoid malignancies between deployed and nondeployed personnel. Interestingly, similar analyses excluding cases diagnosed up to 5 or 10 years after initial deployment were associated with lower risk of developing lymphoid malignancies overall and for several of the individual subtypes. The reasons for this rather unexpected finding are uncertain and merit further investigation. Future research should prospectively follow a cohort of veterans with known carcinogenic exposures for the development of lymphomas and other cancers to determine incidence and characterize the latency from specific exposure to outcomes. Clinical data related to lymphoid malignancy types, such as translocations or genomic aberrations, are important to make prognostic implications. In addition, identifying biomarkers for exposures will be important to use as objective data to limit misclassification in these analyses. Biomarkers and other predictors of disease may also be used to identify high risk individuals, develop screening procedures to diagnose cancers early, and improve health outcomes. These investigations will also be relevant to the civilian population, in view of exposures to wildfires and other environmental toxicants.

## 5. Conclusions

In US veterans who enlisted in the military after 11 September 2001, deployment to the Middle East was not associated with a higher risk of developing lymphoid malignancies compared to those who did not deploy. Excluding cases diagnosed within 5–10 years post-deployment reveals a reduced risk of developing lymphoid malignancies, indicating that later diagnoses may reflect unrelated etiologies.

## Figures and Tables

**Table 1:** Characteristics of veterans who enlisted after 11 September 2001.

Characteristics	Controls (*n* = 3572)	Cases (*n* = 1037)	Total (*n* = 4609)
Age at Diagnosis/Index Age, median years (IQR)	33.5 (27.9–41.9)	35.2 (28.7–45.2)	33.8 (28.1–42.6)
Sex, *n* (%)			
Men	3128 (88)	904 (87)	4032 (87)
Women	444 (12)	133 (13)	577 (13)
Race and Ethnicity			
Non-Hispanic White	2160 (60)	613 (59)	2773 (60)
Non-Hispanic Black	520 (15)	170 (16)	690 (15)
Hispanic	468 (13)	135 (13)	603 (13)
Non-Hispanic API	130 (4)	33 (3)	163 (4)
Non-Hispanic Other	68 (2)	18 (2)	86 (2)
Unknown	226 (6)	68 (7)	294 (6)
Lymphoid category, *n* (%)			
ALL	-	64 (6)	-
BL	-	20 (2)	-
CLL/SLL	-	97 (9)	-
DLBCL	-	146 (14)	-
FL	-	68 (7)	-
HL-AYA	-	261 (25)	-
HL-older	-	34 (3)	-
LPL	-	9 (1)	-
MCL	-	8 (1)	-
MTNKL	-	97 (9)	-
MZL	-	401 (4)	-
NOS	-	79 (8)	-
PCN	-	114 (11)	-

Legend: ALL—acute lymphoblastic lymphoma, API—Asian/Pacific Islander, AYA—adolescent and young adult, BL—Burkitt lymphoma, CLL/SLL—chronic lymphocytic leukemia/small lymphocytic lymphoma, DLBCL—diffuse large B-cell lymphoma, FL—follicular lymphoma, HL—Hodgkin lymphoma, IQR—interquartile range, LPL—lymphoplasmacytic lymphoma, MCL—mantle cell lymphoma, MTNKL—mature T-cell/natural killer cell lymphoma, MZL—marginal zone lymphoma, NOS—not otherwise specified, PCN—plasma cell neoplasm.

**Table 2:** Military characteristics of veterans who enlisted between after September 2001.

Characteristics	Controls (*n* = 3572)	Cases (*n* = 1037)	Total (*n* = 4609)	*p*-Value
Military Branch				0.60
Air Force	414 (12)	113 (11)	527 (11)	
Army	1983 (56)	599 (58)	2582 (56)	
Marine Corps	548 (15)	146 (14)	694 (15)	
Navy	452 (13)	135 (13)	587 (13)	
Other	175 (5)	44 (4)	219 (5)	
Military Rank				0.08
Enlisted (high school)	3119 (87)	926 (89)	4045 (88)	
Officer/warrant officer	246 (7)	69 (7)	315 (7)	
Unknown	207 (6)	42 (4)	249 (5)	
VA OEF/OIF Table				0.89
Yes	2003 (56)	579 (56)	2582 (56)	
No	1569 (44)	458 (44)	2027 (44)	

Legend: OEF/OIF—Operation Enduring Freedom/Operation Iraqi Freedom.

**Table 3:** Exposure and risk of developing lymphoid malignancies.

Model	Total Number of Cases	Total Number of Controls	Odds Ratio	95% Confidence Interval	*p*-Value
All with deployment, rank, branch	1037	3572	1.05	0.89–1.23	NS
All, exclude cases diagnosed 2 years	998	3448	0.95	0.81–1.12	NS
All, exclude cases diagnosed 5 years	902	3107	0.76	0.64–0.90	0.002
All, exclude cases diagnosed 10 years	723	2472	0.41	0.34–0.50	<0.0001

**Table 4:** Exposure and risk of developing individual subtypes of lymphoid malignancies.

Category	Total Number of Cases	Total Number of Controls	Odds Ratio	95% Confidence Interval	*p*-Value
CLL/SLL	97	264	0.93	0.52–1.67	NS
CLL/SLL, exclude 2 years	94	260	0.88	0.49–1.59	NS
CLL/SLL, exclude 5 years	90	252	0.84	0.46–1.52	NS
CLL/SLL, exclude 10 years	73	196	0.41	0.20–0.84	0.02
DLBCL	146	527	0.76	0.49–1.18	NS
DLBCL, exclude 2 years	142	512	0.68	0.44–1.07	NS
DLBCL, exclude 5 years	132	476	0.56	0.35–0.89	0.01
DLBCL, exclude 10 years	111	397	0.33	0.20–0.57	<0.0001
HL-AYA	261	1004	1.09	0.81–1.49	NS
HL-AYA, exclude 2 years	244	943	0.93	0.68–1.28	NS
HL-AYA, exclude 5 years	211	818	0.67	0.47–0.94	0.02
HL-AYA, exclude 10 years	161	623	0.28	0.18–0.45	<0.0001
MTNKL	97	326	0.97	0.58–1.65	NS
MTNKL, exclude 2 years	95	318	0.92	0.54–1.57	NS
MTNKL, exclude 5 years	84	284	0.69	0.39–1.23	NS
MTNKL, exclude 10 years	68	223	0.28	0.13–0.59	0.0008
PCN	114	325	0.98	0.59–1.61	NS
PCN, exclude 2 years	113	325	0.98	0.59–1.61	NS
PCN, exclude 5 years	106	301	0.85	0.51–1.42	NS
PCN, exclude 10 years	83	226	0.44	0.24–0.80	0.007

Legend: CLL/SLL—chronic lymphocytic leukemia/small lymphocytic lymphoma, DLBCL—diffuse large B-cell lymphoma, HL-AYA—Hodgkin lymphoma (adolescent and young adult), MTNKL—mature T-cell/natural killer cell lymphoma, PCN—plasma cell neoplasm.

## Data Availability

Due to the sensitive nature of veteran health information, data will not be made available according to VA policy.

## Abbreviations

The following abbreviations are used in this manuscript:

ALL	Acute lymphoblastic leukemia/lymphoma
API	Asian and Pacific Islander
AYA	Adolescent and young adult
BL	Burkitt lymphoma
CI	Confidence interval
CLL/SLL	Chronic lymphocytic leukemia/small lymphocytic lymphoma
DLBCL	Diffuse large B-cell lymphoma
FL	Follicular lymphoma
HL	Hodgkin lymphoma
HR	Hazard ratio
ICD-O-3	International Code of Diseases, Oncology, Version 3
IQR	Interquartile range
IRB	Institutional Review Board
LPL	Lymphoplasmacytic lymphoma
MCL	Mantle cell lymphoma
MTNKL	Mature T-/NK-cell lymphoma
MZL	Marginal zone lymphoma
NOS	Not otherwise specified
OEF	Operation Enduring Freedom
OIF	Operation Iraqi Freedom
OR	Odds ratio
PCN	Plasma cell neoplasm
VA	Veteran Affairs
VACCR	VA Central Cancer Registry
VINCI	VA Informatics and Computing Infrastructure
